# Restoration of endodontically treated cracked maxillary teeth: A case series

**DOI:** 10.1002/ccr3.2391

**Published:** 2019-09-01

**Authors:** Hossein Ali Mahgoli, Mahnaz Arshad, Kamran Rasouli

**Affiliations:** ^1^ Dental Research Center, Dentistry Research Institute Tehran University of Medical Sciences Tehran Iran; ^2^ Department of Prosthodontics, School of Dentistry Tehran University of Medical Sciences Tehran Iran; ^3^ International Campus, School of Dentistry Tehran University of Medical Sciences Tehran Iran

**Keywords:** cracked tooth syndrome, endodontically treated teeth, nonvital teeth

## Abstract

Cracks adversely affect the prognosis and survival of the teeth. Thus, the possibility of presence of crack should be considered after endodontic treatment and before the initiation of periodontal/prosthetic treatments. Attempts must be made to restore cracked teeth with efficient restorative materials to increase their survival and prevent additional costs.

## 1 INTRODUCTION

Longitudinal tooth fractures can be divided into five groups in terms of severity: (I) craze lines, (II) fractured cusp, (III) cracked tooth, (IV) vertical root fracture, and (V) split tooth. The greater the extension of crack or fracture line from the coronal toward the apical region, the greater the severity of trauma and damage to the tooth structure would be. This type of fracture may occur in all teeth and has a high prevalence. It occurs as the result of application of wedging occlusal forces or some dental procedures.^1^, ^2^ In this paper, we mainly focus on longitudinal fractures causing cracks, which are classified under group III. The location of type III fractures is mainly in the tooth crown, and these cracks extend from the crown toward the root with variable depths. They occur in mesiodistal direction in 81.1% of the cases.^3^ The crack origin is in the occlusal surface, and it often occurs due to parafunctional habits or in teeth with compromised tooth structure. The signs and symptoms of cracked teeth may widely vary. For crack identification, a hard object (such as a tongue blade) can be placed between the teeth and the patient is asked to bite on it. This can cause an unpleasant sensation such as pain in the respective tooth. Identification and diagnosis of a cracked tooth are challenging. Diagnosis of a cracked tooth is generally made based on clinical symptoms such as presence of local pain on biting, hypersensitivity to cold and pain upon removal of pressure from the tooth. The patient feels pain in the respective tooth when bites on a hard object and also when opens his mouth and removes the pressure from the tooth. However, for more efficient detection of cracks, it would be better to remove the existing restoration and perform transillumination, staining, and wedge segment tests, which can be unpleasant for the patients. Isolated/narrow periodontal probing, biting test, and magnification loupes can also be used for this purpose. The treatment often consists of root canal therapy according to the pulpal and periradicular diagnosis and full cuspal coverage with bonded restorative materials. However, such teeth often have questionable to poor prognosis. A cracked tooth is defined as presence of an incomplete crack originating from the tooth crown and extending subgingivally in mesiodistal direction.^2^, ^4^, ^5^

According to Seo et al, cracks often occur in restored teeth especially those with mesio‐occluso‐distal amalgam and inlay restorations. The reason is attributed to (I) presence of sharp angles in the prepared cavity since sharp angles are the sites of stress accumulation and potentially increase the risk of tooth fracture and (II) the mismatch between the coefficients of thermal expansion of restorative materials and tooth structure, which is another reason for crack formation. However, some others believe that cracks occur with the same frequency in sound and restored teeth.^6^

In general, it may be stated that the management of such teeth may vary depending on the size and depth of cracks such that when the patient's chief complaint is mild pain or hypersensitivity when biting, banding of tooth can result in pain relief. The banding can be replaced with a prosthetic crown. However, when the crack involves dental pulp, the treatment plan changes to root canal therapy and post and core fabrication. Berman and Kuttler stated that necrotic teeth with longitudinal cracks should be preferably extracted. Also, teeth with deep periodontal pockets have a poor prognosis. Sim et al reported 92% success rate following root canal treatment of teeth with cracks involving their pulp.^7^

In this study, we focus on restored teeth with cracks involving the pulp chamber floor. Evidence shows that such teeth have a poor prognosis especially posterior teeth such as maxillary and mandibular molar teeth in which, the pulp chamber floor has lower thickness and strength due to the presence of furcation. In such teeth, crack propagation causes root splitting and exposure of periodontal ligament to oral microbial flora and saliva.^7^

This paper aims to introduce a technique for restoration and preservation of teeth with transverse cracks involving the pulp chamber floor.

## 2 CASE I

**Figure 1:**
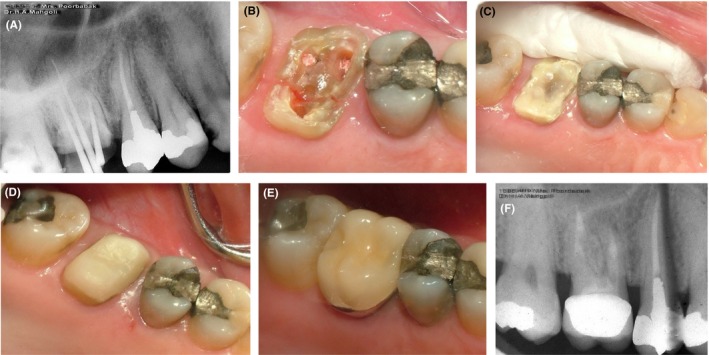
Case I, A, PA radiograph of the maxillary right first molar; B, Cracks are visible in the pulpal floor after restoration removal; C, Application of Panavia cement and allowing time for cement setting (a minimum of 24 h); D, Composite build‐up and core fabrication; E, Final restoration cemented with glass ionomer cement; F, Follow‐up radiograph taken at 18 mo

Our first case was a 49‐year‐old male complaining of pain in his maxillary right first molar. The patient presented to the Prosthodontics Department of Tehran University of Medical Sciences with a chief complaint of moderate sharp pain in the respective tooth. The patient recalled that the pain started two weeks earlier following accidental biting on a hard object when eating, which resulted in fracture of a part of his tooth. The patient's medical history was unremarkable. The patient's dental history revealed self‐reported bruxism and use of night‐guard for the past 7 months. Wear of incisal edges of the anterior teeth and the posterior cusp tips due to bruxism was evident. The patient's oral hygiene status was moderate, and dental plaque and calculus were found in lower amounts on the lingual surfaces of his mandibular anterior teeth. Extraoral examination confirmed symmetry of the face, and no specific problem was detected. The patient had strong masticatory muscles, which along with bruxism, could have increased the risk of cracking of teeth. After clinical examination, a periapical (PA) radiograph was obtained of the respective tooth (Figure 1A), which did not reveal any sign of crack. The respective tooth had previously undergone endodontic treatment and had an amalgam restoration. However, the restoration had a poor quality. We decided to remove the restoration and then decide on the treatment plan. After removal of secondary caries, the crack line appeared in the pulp chamber floor under the amalgam restoration (Figure 1B). Carious tissue was removed with a round bur and low‐speed hand‐piece to better reveal the crack line. After caries removal, the crack line appeared, which had a mesiodistal orientation and had almost resulted in splitting of buccal and palatal roots. Probing depth more than 3 mm was not detected. There was no active bleeding either.

Since a great portion of the cavity walls was lost and could not be restored, the treatment plan included post and core fabrication and metal‐ceramic crown placement.

After administration of anesthetic agent, the surface was etched with phosphoric acid in order to increase the retention of cement. The tooth was then disinfected with chlorhexidine. Panavia self‐cure cement (Panavia F, Kuraray Co) was applied for bonding of the broken tooth segments such that the cement was applied over the crack line. Following slight setting of the cement, calcium hydroxide and temporary dressing (Cavit; 3M ESPE) were applied (Figure 1C, 1D). To allow complete setting of the cement, the rest of treatment was performed in the next session. In the second session, the post and core restoration was fabricated. The palatal canal was emptied by Gates‐Glidden drills (Dentsply, Maillefer) for the fabrication of post and core. Using a peeso reamer (Dentsply, Maillefer), the canal was completely shaped. The post and core restoration was fabricated using glass fiber‐reinforced composite material. The tooth was then prepared for a metal‐ceramic crown. Gingival retraction cord was placed, and a final impression was made using double‐mix two‐stage technique for the fabrication of final cast. In the next session, the seating of crown was evaluated and after removing the pressure points and occlusal adjustment, the crown was permanently cemented using glass ionomer cement (GC, International crop; Figure 1E). The patient was followed up at 6 months and annually thereafter. The follow‐up radiograph obtained at 18 months revealed no problem related to the respective tooth (Figure 1F).

## 3 CASE II

**Figure 2:**
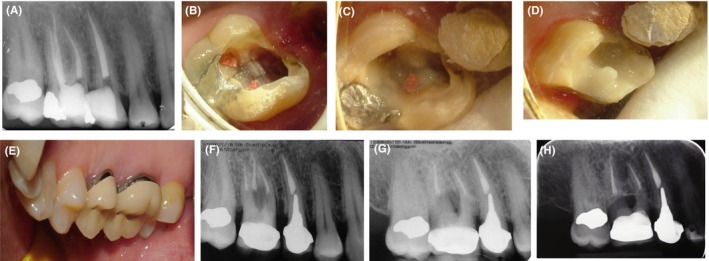
Case II, A, PA radiograph of the maxillary right first molar; B, Crack lines visible in the pulp chamber floor; C, Restoring the crack lines in the pulp chamber floor with Panavia; D, Glass ionomer cement applied as base; E, Final crown fabricated for the patient; F, Final periapical radiograph; G, Follow‐up radiograph taken at 28 mo; H, Follow‐up radiograph taken at 10 y

Our patient was a 52‐year‐old female presenting with the chief complaint of pain and hypersensitivity of her maxillary right first molar. The patient's medical history was unremarkable. The patient's dental history revealed bruxism. The patient had a night‐guard but was not using it. Intraoral clinical examination revealed wear of occlusal surfaces of the teeth due to bruxism. Extraoral clinical examination was normal. The respective tooth gave a mild response to percussion and bite tests. A PA radiograph was obtained to assess the periodontium (Figure 2A). The tooth did not have any PA or furcal lesions and had been endodontically treated. Local anesthesia was administered, and the restoration was removed. The crack lines were evident in the furcal floor (Figure 2B). Probing depth more than 3 mm was not detected. There was no active bleeding either.

The crack line and the cavity walls were rinsed with chlorhexidine to eliminate bacteria. After acid etching, the crack line was covered with Panavia resin cement. Glass ionomer cement was used as a base (Figure 2C and 2). The rest of treatment was performed in the next session to allow polymerization of cement. The final treatment plan was placement of a metal‐ceramic crown. In the next session, the tooth was restored with composite resin. Then, it was prepared, the core and the prosthetic crown were fabricated and the crown was cemented in the next session (Figure 2E). The final PA radiograph (Figure 2F) and the follow‐up radiograph taken after 28 months (obtained at the third follow‐up session) revealed no problem related to the respective tooth (Figure 2G). The 10‐year follow‐up radiograph showed no problem either (Figure 2H).

## 4 CASE III

**Figure 3:**
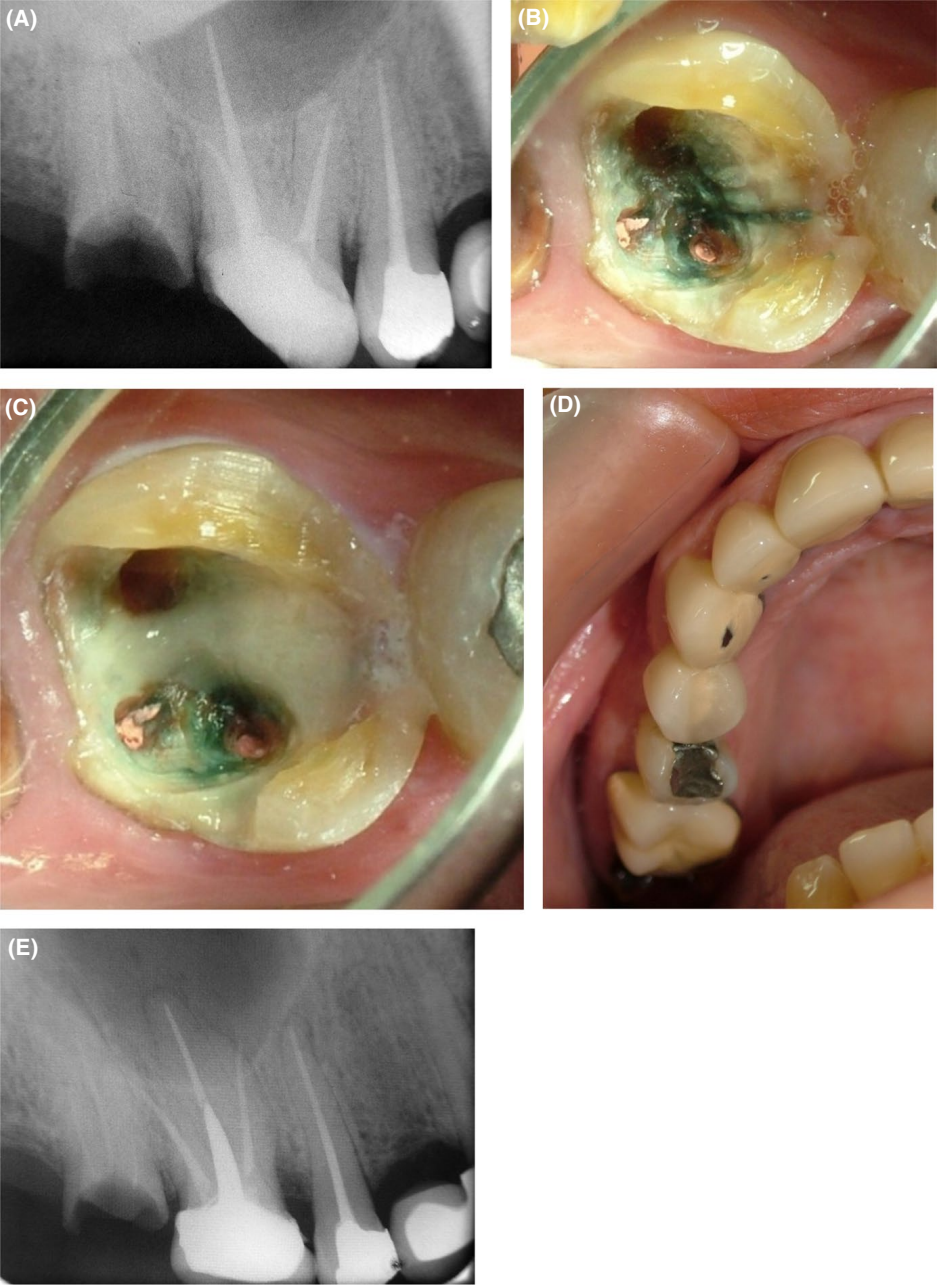
Case III A, PA radiograph of the maxillary right first molar; B, Crack lines are visualized by staining of the pulp chamber floor (transillumination); C, Covering the crack lines with Panavia cement; D, Fabrication of final prosthetic crown; E, Final radiograph

Our third patient was a 32‐year‐old male complaining of pain and hypersensitivity of his maxillary right first molar. The patient reported root canal treatment of the respective tooth 4 months earlier. The tooth had an amalgam restoration. He stated that the restoration was overcontoured at first and his pain did not resolve after repeated dental visits. The patient responded positively to percussion and bite tests, which indicated periodontal inflammation. Extraoral clinical examination was normal, and the patient was systemically healthy. A PA radiograph was obtained, which showed periodontal ligament widening around the apex probably due to overcontouring of the tooth and traumatic occlusion (Figure 3A). PA radiograph revealed no crack in the respective tooth. Occlusal adjustment was performed to remove pressure from the tooth. Two weeks were allowed in order for the pain and inflammation to subside, and the patient was scheduled for a follow‐up. After two weeks, the patient was clinically examined. His chief complaint was pain upon chewing. Another PA radiograph was obtained, which showed that the periodontal ligament widening had been resolved. Thus, the patient probably had a crack. Since the crack was not visible on the PA radiographs, the restoration was removed. After removal of the restoration and cleaning of the pulp chamber floor, a mesiodistal fracture line appeared as a bold line between the orifices by transillumination (Figure 3B). The treatment was performed as described for the above‐mentioned two cases (restoration of crack line with Panavia and fabrication of post and core restoration for the tooth; Figure 3C, 3D, 3E).

## 5 CASE IV

**Figure 4:**
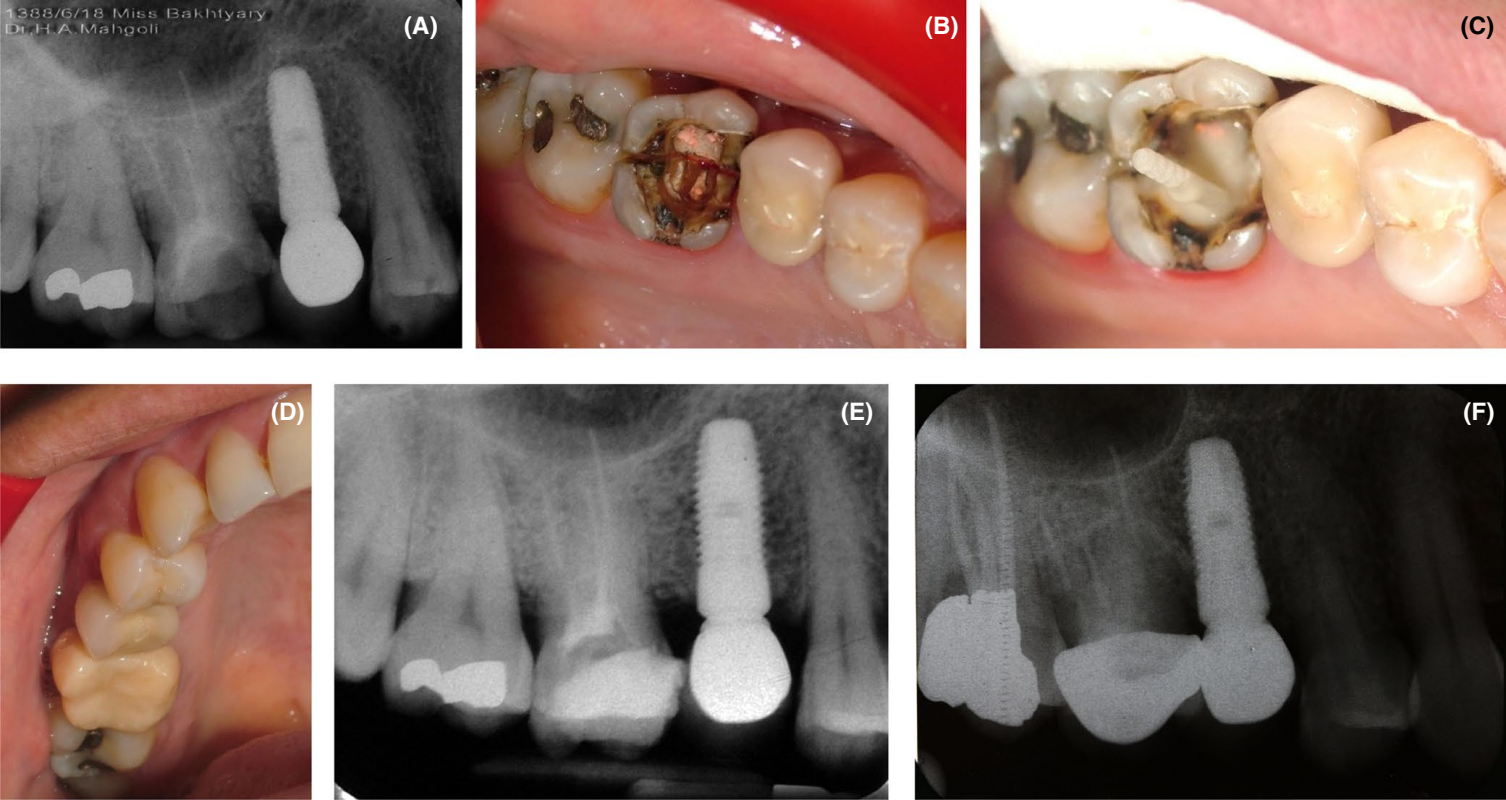
Case IV, A, PA radiograph of the maxillary right first molar; B, A mesiodistal crack in the pulp chamber floor is visible; C, Post and core fabrication; D, Final crown fabricated for the respective tooth; E, Final radiograph taken after crown delivery; F, Follow‐up radiograph taken after 8 y

Our patient was a 35‐year‐old female presenting with pain and hypersensitivity in the right posterior maxilla. The pain was severe and intermittent and intensified by chewing on hard objects. The patient's medical history was unremarkable. Her maxillary first molar had been endodontically treated one year earlier and had an amalgam restoration, which was extensive and had a poor quality. PA and panoramic radiographs were obtained, which did not reveal any crack or PA lesion (Figure 4A). The tooth was not sensitive to percussion. The differential diagnosis included fracture and crack. To reach an accurate diagnosis, amalgam restoration was removed. An extensive crack line was noted in the pulp chamber floor, which had a mesiodistal direction, splitting the tooth at the furcation (Figure 4B). Post (fiber post) and core and crown were fabricated, and the patient was managed as reported for previous cases (Figure 4C and 4D). A final PA radiograph was also obtained (Figure 4E), and the patient was scheduled for a follow‐up. A PA radiograph was obtained at each follow‐up session (Figure 4F). The patient has been asymptomatic so far. The 9‐year follow‐up radiograph showed no problem related to this tooth (Figure 4F).

## 6 DISCUSSION

As mentioned earlier, management of cracked teeth may vary depending on the size and severity of cracks. When the pulp chamber is involved, the tooth would require root canal therapy and post and core fabrication. Cracks may develop as the result of impact and trauma, biting on a hard object or presence of an inappropriate, overcontoured restoration, applying excessive pressure to the tooth and creating a wedging force.^3^

In general, PA radiography is not helpful for detection of mesiodistal cracks in the pulp chamber floor because they are tiny and not detectable due to superimposition of buccal and lingual surfaces. In general, PA radiography mainly reveals cracks that are oriented parallel to the direction of the radiated X‐ray beam. Thus, cross‐sectional radiographs such as CBCT scans are recommended for such cases, which can visualize the crack in the sagittal and axial planes.^3^

The teeth are more susceptible to cracks following endodontic treatment. Thus, correct fabrication of post and core and crown can help decrease the frequency of tooth fractures and cracks and increase the success rate of restorations.

Type of cement is an important parameter determining the success of treatment. We used Panavia along with glass ionomer cement due to the similarity of modulus of elasticity of Panavia cement to that of dentin. The modulus of elasticity is an important parameter in assessment of cements, and selecting a cement with a modulus of elasticity similar to that of dentin maximizes the efficacy of cement and increases the compatibility of cement with dentin. On the other hand, it would result in better stress distribution in the cement and dentin and would prevent stress accumulation in the cement at the crack site.^8^

Evidence shows that presence of cracks in the pulp chamber floor would significantly affect the prognosis of the tooth. Presence of cracks in the pulpal floor and their extension to areas lower than the alveolar bone crest would deteriorate the prognosis (from poor to hopeless) and may necessitate tooth extraction. Glass ionomers and adhesives have been previously used for bonding of broken segments of teeth as in cusp fractures when the crack has not invaded the cementoenamel junction.^3^

In selection of our suggested technique for management of cracked teeth, it is important that the teeth do not have any active bleeding or periodontal pocket in order to allow the Panavia cement to easily flow into the crack.

## 7 CONCLUSION

The main advantage of our suggested technique compared with previous ones is that it enables the restoration of mesiodistal cracks in the pulpal floor and furcation area without necessitating tooth extraction. Also, it impedes crack propagation and splitting of the tooth. Our patients were followed up for 10 years (annually), and their clinical examination and serially taken radiographs did not reveal any sign of reappearance of cracks, hypersensitivity to bite forces or PA lesions, and all patients were satisfied with the treatment outcome.

## CONFLICT OF INTEREST

The authors have no conflict of interests to declare. The authors alone are responsible for the content and writing of the paper.

## AUTHOR CONTRIBUTIONS

MA: Conceived the idea, performed the treatment, collected the data, and submitted the paper. HM: Conceived the idea and obtained the images. KR: Wrote the manuscript, was involved in project management, and collected the data.
